# Beyond standard English: how Global Englishes enhances creativity, reduces language anxiety, and increases willingness to communicate

**DOI:** 10.3389/fpsyg.2025.1611630

**Published:** 2025-06-18

**Authors:** Asep Budiman, Xinyu Liu

**Affiliations:** ^1^Department of English Language and Literature, Hunan Normal University, Changsha, China; ^2^Department of English Language Education, University of Nahdlatul Ulama Purwokerto, Purwokerto, Indonesia

**Keywords:** Global Englishes, language teaching, second language acquisition, creativity, language anxiety, willingness to communicate, learner psychology, multilingual education

## Abstract

This article explores how the Global Englishes Language Teaching (GELT) paradigm promotes learner psychology by enhancing creativity, reducing language anxiety, and increasing willingness to communicate (WTC) in second or foreign language (L2) contexts. Drawing on key psychological and pedagogical theories alongside empirical research, the article argues that GELT offers a transformative alternative to traditional English language teaching (ELT) by validating diverse English varieties, reframing errors as communicative resources, and fostering learner agency. Through an interdisciplinary synthesis, the study demonstrates how GELT aligns with dynamic, learner-centered approaches to second language acquisition (SLA), enabling learners to express themselves more authentically, confidently, and creatively. The findings suggest that embracing Global Englishes not only supports linguistic competence but also nurtures emotional well-being and communicative autonomy, offering valuable insights for teachers, curriculum designers, and policymakers in today’s increasingly multilingual educational contexts.

## 1 Introduction

English has evolved into a global lingua franca, used by millions of non-native speakers across diverse sociocultural and professional contexts. This transformation has led to growing critiques of native-speaker norms in ELT, which have traditionally prioritized grammatical precision, native-like pronunciation, and adherence to standardized British or American varieties (Jenkins, 2015; Galloway, 2017). While these norms have long shaped curricula, assessments, and classroom practices, they often fail to reflect how English is actually used in real-world, multilingual communication. As a result, learners may feel pressure to conform to unrealistic standards, potentially impeding their communicative confidence and engagement.

In response, the Global Englishes paradigm has emerged as a critical and inclusive alternative. Building on theoretical models such as World Englishes (WE), English as an International Language (EIL), English as a *Lingua Franca* (ELF), translanguaging, and multilingualism, Global Englishes advocates for a pluralistic view of English that embraces variation, intelligibility, and intercultural adaptability (Kohn, 2022; Schreiber and Jansz, 2025). This approach challenges the ideological dominance of native-speakerism by recognizing that English is constantly shaped by its global users—many of whom are multilingual speakers from diverse linguistic backgrounds (Crystal, 2003). Despite the momentum of this paradigm, however, many ELT practices remain anchored in monolithic norms that marginalize linguistic diversity and overlook learners’ psychological needs (Yunhua and Budiman, 2024).

While previous research has explored the sociolinguistic and pedagogical implications of Global Englishes, there has been limited attention to its psychological dimensions. Specifically, the potential of GELT to support learners’ creativity, reduce language anxiety, and increase WTC remains underexplored. These three constructs are central to SLA and significantly influence learners’ motivation, confidence, and performance. Yet, in most GELT discussions, they are only tangentially addressed, if at all.

This perspective article addresses a critical gap in the Global Englishes literature: the limited attention to psychological variables that shape learner engagement and success. Drawing from established theories and empirical studies in applied linguistics and educational psychology, we propose a conceptually grounded interpretation of how GELT can enhance three key psychological outcomes—creativity, language anxiety, and willingness to communicate. Rather than presenting new empirical data or a systematic literature review, this article offers a parsimonious framework—GELT for psychological benefits—that synthesizes sociolinguistic principles and psychological constructs. Our aim is to stimulate dialogue and future empirical research by demonstrating how GELT can contribute not only to linguistic competence, but also to learner well-being, autonomy, and communicative agency in global classrooms.

## 2 Global Englishes language teaching

Traditional ELT has long served as the foundation of language education in many global contexts, offering structured methodologies grounded in well-established theories of language acquisition (Klee et al., 1986; Barnard et al., 2002). Emphasizing linguistic accuracy, standard pronunciation, and native-like fluency, this approach has proven effective in developing learners’ grammatical proficiency and formal command of the language. Standardized curricula and assessment frameworks have also contributed to a sense of consistency and predictability for both teachers and learners (Terrell and Brown, 1981).

However, despite its merits, traditional ELT has been increasingly criticized for its limited responsiveness to the psychological and communicative needs of learners in today’s globalized world. A major concern is its tendency to elevate native-speaker norms, particularly those associated with British and American English, as the primary models of correctness. This narrow orientation often marginalizes the rich diversity of English varieties used by non-native speakers globally, reinforcing linguistic hierarchies that can undermine learners’ confidence and communicative competence (Holliday, 2006; Galloway, 2013).

On the other hand, GELT offers a more inclusive and psychologically empowering alternative. GELT reframes English as a shared global resource, not the property of any single group, and emphasizes the legitimacy of diverse Englishes shaped by their global users (Rose et al., 2021). Instead of enforcing a singular linguistic standard, GELT values variation, fluidity, and adaptability, encouraging learners to view their English use as both legitimate and meaningful.

This pedagogical shift has important psychological implications. By validating diverse English varieties and prioritizing communicative effectiveness over native-like accuracy, GELT reduces learners’ language anxiety—a key barrier in SLA. Learners are less fearful of making mistakes when the classroom environment recognizes multiple ways of speaking and expressing meaning (Dewaele and Alfawzan, 2018; Dewaele and MacIntyre, 2024; Gong et al., 2025). This reduction in anxiety fosters greater WTC, a known predictor of language success, as learners feel more confident initiating conversations and contributing to class discussions (Cao and Wei, 2019; Peng and MacIntyre, 2025). Moreover, GELT creates space for creativity in language use, as it reframes English as a dynamic and context-sensitive resource rather than a static set of prescriptive rules. Learners are encouraged to experiment with different expressions, blend linguistic resources, and engage in translingual practices that reflect their lived experiences and identities (Pennycook, 2006; Pang and Li, 2024).

While traditional ELT may continue to serve particular instructional goals—especially those focused on formal writing, test preparation, or standardized language use—its limitations are increasingly evident in multicultural and multilingual settings. GELT responds to these limitations by promoting intercultural communicative competence, affirming learner identities, and reducing affective barriers to language development. Emerging research supports the psychological effectiveness of GELT, particularly in classrooms where inclusivity, emotional safety, and learner autonomy are prioritized (Wang, 2024).

**Figure 1:**
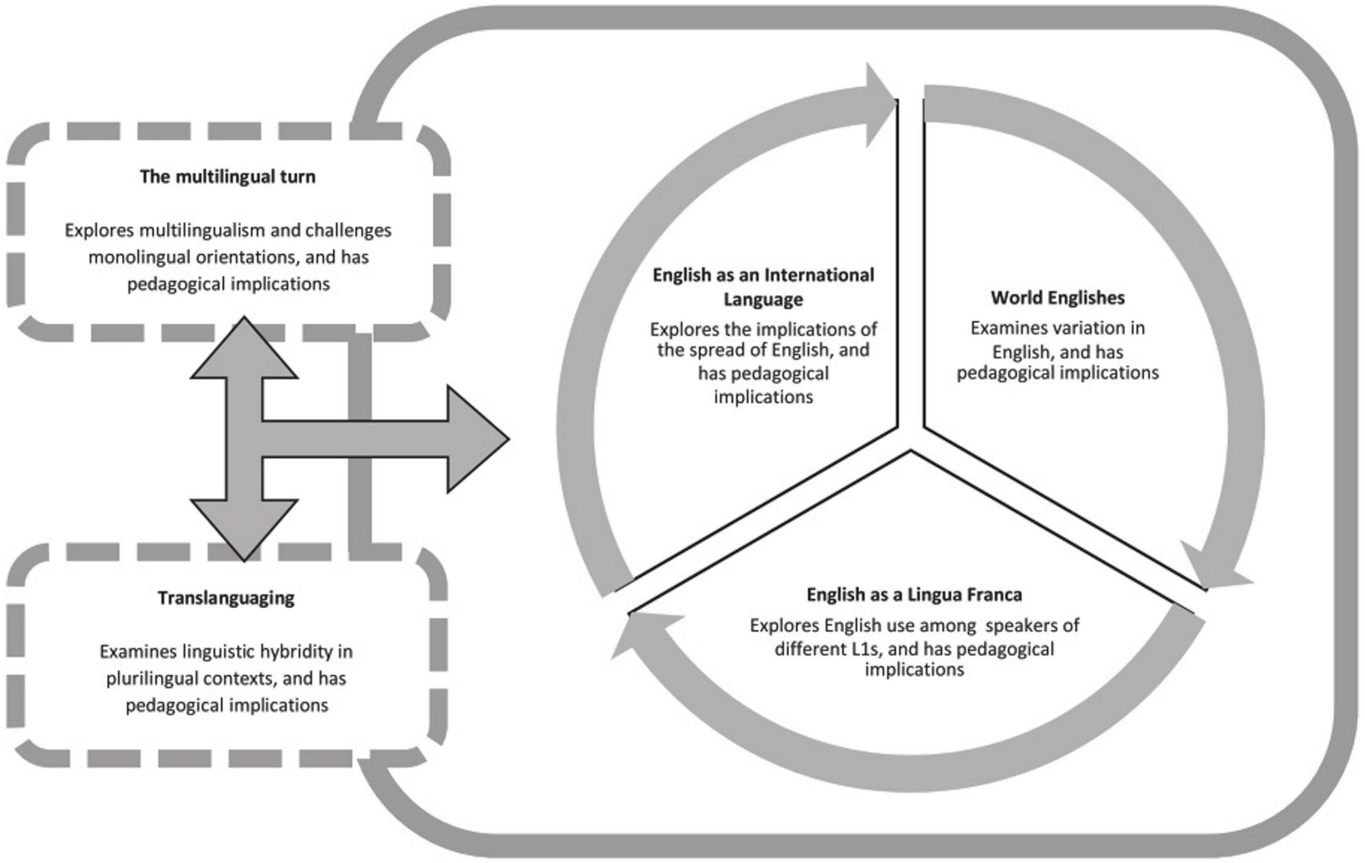
Global Englishes: an inclusive paradigm. Source: Rose and Galloway (2019).

Ultimately, GELT represents a paradigm shift in language education—one that centers learner psychology, embraces linguistic diversity, and redefines success in communication not by conformity to native-speaker norms, but by adaptability, intelligibility, and confidence. By preparing learners to engage meaningfully in a globalized, multilingual world, GELT provides the tools for psychologically healthy, creative, and empowering language learning experiences. The collaborative vision underpinning these developments is unified under the umbrella term of Global Englishes, as illustrated in Figure 1.

## 3 Discussion

### 3.1 GELT framework for psychological benefits

ELT is increasingly being reimagined through the lens of Global Englishes, with a growing emphasis on preparing learners for interaction in linguistically diverse, intercultural settings. The GELT framework, as introduced by Galloway (2013) and refined by Galloway and Rose (2015), proposes a paradigm shift toward: (1) integrating WE and ELF into language curricula; (2) valuing multilingualism as a resource; (3) raising awareness of Global Englishes; (4) incorporating ELF-informed communication strategies; (5) embracing cultural fluidity; and (6) diversifying teacher recruitment practices to reflect global English ownership.

In this perspective article, we build on this foundational GELT framework by proposing a psychologically expanded interpretation that synthesizes key sociolinguistic principles with affective variables in SLA—namely creativity, language anxiety, and WTC. This proposal is grounded in a conceptual integration of empirical findings and theoretical insights from both applied linguistics and educational psychology. Rather than a systematic review, our aim is to offer a parsimonious conceptual model that highlights GELT’s under-explored potential to enhance psychological well-being and communicative autonomy.

This framework is guided by two key assumptions: (1) Language learning is not only cognitive, but deeply affective and identity-related. (2) Pedagogical environments that affirm linguistic diversity are more likely to foster learner confidence, risk-taking, and engagement.

Thus, our contribution lies in mapping existing GELT dimensions to their psychological impacts—providing a novel theoretical lens for understanding how inclusive pedagogies support learner flourishing. This addresses an important gap in the literature, where the affective benefits of GELT remain conceptually underdeveloped. In line with Simon’s (2002) argument for theoretical economy, the framework presented here maintains interpretability by focusing on a small number of variables with robust empirical support and pedagogical relevance.

The following table illustrates how each core component of GELT may support one or more psychological outcomes:

### 3.2 Creativity as a psychological catalyst in GELT

A key contribution of the GELT for psychological benefits framework is the repositioning of creativity from a peripheral trait to a central pedagogical objective in language learning. Creativity is not merely an aesthetic bonus, but a critical cognitive and emotional resource that empowers learners to adapt language to real-world communicative demands. Traditional ELT practices, with their emphasis on standardized norms and error avoidance, often suppress learners’ willingness to experiment with language. GELT, by contrast, promotes an environment where linguistic innovation, identity expression, and multilingual resourcefulness are not only permitted but encouraged.

Within GELT, English is conceptualized not as a static system to be mastered, but as a flexible, adaptive tool that responds to varied sociolinguistic contexts. This reconceptualization aligns with psychological research showing that creativity flourishes in low-anxiety, high-autonomy settings, where learners are invited to take risks, explore meanings, and draw from their full linguistic repertoires. As illustrated in Table 1, several dimensions of GELT—such as fluid norms, inclusive ideology, and multilingual orientation—directly support these conditions, thereby activating creativity as a psychological and pedagogical catalyst.

**Table 1:** GELT framework and its psychological impacts on learners.

Dimensions	GELT	Psychological Impact
Target interlocutor	All English users	Boost WTC by normalizing diverse interaction contexts
Ownership	Global	Enhances learner agency and ownership over language
Target culture	Fluid cultures	Supports cultural empathy and reduces anxiety in diverse settings
Norms	Diverse, flexible, and multiple forms	Encourages creative expression; reduces performance pressure
Teachers	Qualified, competent teachers (same and different L1s)	Improve confidence by removing native speaker bias
Role model	Expert users	Provides relatable, attainable role models; supports self-efficacy
Source of materials	Salient English-speaking communities and contexts	Reinforces learner identity and motivation through relevance
Other languages and cultures	Seen as a resource as with other languages in their linguistics repertoire	Reduces anxiety and promotes pride in multilingual identity
Needs	Globally defined	Aligns learning goals with learners’ realities, enhancing motivation
Assessment criterion	Communicative competence	Shifts focus from error to meaning, lowering anxiety
Goals of learning	Multicompetent users	Fosters creativity and engagement with pluralistic norms
Ideology	Underpinned by an inclusive Global Englishes perspective	Validates learners’ voices, supporting confidence and belonging
Orientation	Multilingual/translingual	Encourages flexible communication, enhancing WTC and creativity

Adapted from: Rose and Galloway (2019).

This theoretical position is supported by evidence from Global Englishes classrooms. For example, Rosenhan and Galloway’s (2019) study of poetry writing tasks in a GELT-informed EAP curriculum showed how creative tasks allowed learners to explore their linguistic identities, challenge dominant norms, and develop voice. These findings align with earlier work by Baker (2001) and Baker and Eggington (1999), who demonstrated the role of bilingual creativity in reshaping linguistic norms and fostering multidimensional expression in World Englishes contexts. Together, they underscore our argument that creativity is not an ancillary benefit but a psychologically empowering force that strengthens engagement, confidence, and agency.

Moreover, creativity supports learners’ motivation and communicative competence. When learners feel authorized to shape language to meet their needs, they become more willing to communicate and less anxious about correctness. As they develop a sense of ownership over language, they also internalize the belief that they are legitimate, capable English users—a belief foundational to resilience and success in SLA.

GELT fosters creativity not as a spontaneous occurrence but as an intentional outcome of inclusive, learner-centered pedagogy. It transforms classrooms into collaborative spaces where language is co-constructed, identities are explored, and learners are empowered to innovate. This perspective highlights creativity as a core psychological mechanism through which GELT enhances language learning outcomes.

### 3.3 Language anxiety: from deficit to resilience in GELT

A major contribution of the GELT for psychological benefits framework lies in its redefinition of how language anxiety is addressed in second language learning. Rather than treating anxiety as a barrier to be minimized solely through technical instruction, GELT frames it as a socially and ideologically constructed phenomenon—one that can be transformed through inclusive pedagogy. Traditional ELT models, which emphasize native-like accuracy and frequent correction, often trigger debilitating anxiety in learners, especially when their performance is judged against unattainable native-speaker ideals (Horwitz, 2016; Amorim, 2013; Sung, 2024).

GELT offers a paradigm shift by foregrounding intelligibility over perfection, and identity affirmation over deficit correction. As outlined in Table 1, its emphasis on flexible norms, global ownership of English, and multilingual resources creates an affective climate in which learners can take risks without fear of failure. This reduction in evaluative pressure directly supports lower levels of language anxiety, a factor consistently linked to improved participation and fluency.

Importantly, this framework aligns with contemporary views of anxiety as dynamic and context-sensitive. Studies using the idiodynamic method have shown that language anxiety fluctuates in real time depending on social context, task demands, and perceived self-competence (MacIntyre and Serroul, 2014; Gregersen et al., 2016). GELT’s learner-centered and adaptive orientation allows teachers to respond to these fluctuations flexibly, promoting greater emotional attunement in teaching practice.

Research also supports the transformative potential of GELT-based instruction. Learners exposed to varied English varieties and teachers who embrace pluralism report decreased fear of negative evaluation and increased willingness to speak (Köylü and Tracy-Ventura, 2022; Mei, 2024). In such classrooms, language anxiety becomes manageable—not eliminated, but redirected into constructive engagement, as suggested by Dewaele and MacIntyre (2016), who found that learners often thrive when anxiety is balanced with curiosity, enjoyment, and meaning-making.

Critically, GELT also reframes learner identity. By validating non-standard accents and multilingual expression, it dismantles the internalized belief that learners must “fix” their English to be taken seriously. Instead, they are encouraged to see themselves as competent, legitimate users of English, which fosters a deeper sense of psychological safety and resilience (Ke, 2016; Saito et al., 2025).

GELT does not simply reduce anxiety—it recasts it. It moves from a deficit-based understanding to a growth-oriented, emotionally responsive model of language learning. This conceptual shift is central to the framework’s contribution: anxiety becomes a signal, not a flaw, and its productive management becomes a pathway to confident, empowered communication in global contexts.

### 3.4 Willingness to communicate: empowering voice in Global Englishes

One of the most distinctive psychological contributions of the GELT for psychological benefits framework lies in its redefinition of WTC as a socially and ideologically responsive construct. While WTC has been recognized as a key predictor of successful SLA (MacIntyre et al., 2016), it is often undermined in traditional ELT contexts where learners feel constrained by native-speaker models and high-stakes performance expectations. These conditions create a communicative climate of risk-aversion, discouraging participation even among linguistically capable learners (Henry et al., 2024).

The GELT framework addresses this problem by centering communicative safety, legitimacy, and agency. As outlined in Table 1, it promotes learner confidence by shifting evaluation away from native-like accuracy toward successful communication in pluralistic, intercultural contexts. This reconceptualization reduces the threat of negative evaluation and affirms the legitimacy of diverse Englishes (Cao and Wei, 2019; Tarp, 2021). In this way, GELT transforms WTC from a passive trait into an active pedagogical outcome—something that can be nurtured through inclusive design, identity validation, and reduced social pressure.

Central to this transformation is the role of self-perception. Learners immersed in deficit-based environments often internalize the idea that their English is not “good enough,” regardless of communicative ability (Lee et al., 2013). GELT subverts this by fostering linguistic self-confidence and positioning variation as a norm rather than a deviation. This shift empowers learners to see themselves as legitimate speakers and encourages them to initiate communication without fear of linguistic inferiority (Geoghegan, 2017; Reynolds and Yu, 2018).

Moreover, the framework responds to empirical findings from study abroad and ELF contexts that show how WTC fluctuates depending on interpersonal dynamics and cultural orientation. In Humphries et al.’s (2023) longitudinal study, Japanese learners reported significantly higher WTC when communicating with other L2 speakers than with native anglophones, suggesting that perceived communicative equality plays a critical role. Similarly, Normann (2021) found that intercultural group interactions during short Erasmus+ exchanges increased learners’ communicative initiative and metacognitive awareness—two outcomes strongly aligned with GELT principles.

By fostering psychologically safe classrooms, GELT creates spaces where learners feel entitled to speak—not merely permitted. These conditions are particularly important given WTC’s high sensitivity to affective variables such as anxiety, self-efficacy, and identity salience. Learners consistently report increased confidence, willingness to take risks, and greater communicative spontaneity in classrooms that normalize linguistic variation and value intelligibility over correctness (Feng et al., 2025).

Finally, GELT enhances learners’ sociolinguistic awareness, enabling them to understand English as a fluid and adaptive resource shaped by negotiation and context. This broader perspective supports the development of a global communicative mindset and reinforces the psychological foundations of WTC—namely, voice, agency, and confidence. Through this lens, WTC is not just an individual disposition but a relational, teachable, and environmentally shaped capacity. The GELT framework thus contributes a crucial insight to SLA theory: that willingness to communicate can be cultivated—not merely predicted—through inclusive pedagogical design and identity-affirming practice.

## 4 Conclusion

This article has proposed a conceptual framework—GELT for psychological benefits—that integrates Global Englishes pedagogy with key affective constructs in second language acquisition: creativity, language anxiety, and willingness to communicate. By moving beyond the restrictive paradigms of traditional ELT, the framework argues that GELT offers more than a sociolinguistic update—it delivers essential psychological support for learners navigating diverse communicative realities. Through the validation of multilingual identities, the reframing of errors as resources, and the redefinition of communicative success, GELT enables learners to speak with greater confidence, less fear, and more expressive freedom.

However, this perspective is not without limitations. As a conceptual article, it does not include empirical testing of the proposed framework, and its psychological claims are grounded in secondary synthesis rather than primary data. Moreover, while the framework draws from diverse research traditions, further theoretical refinement is needed to ensure parsimony and interdisciplinary coherence (Simon, 2002). Future empirical studies—both qualitative and quantitative—are necessary to test the validity and classroom applicability of the framework across different cultural and institutional contexts.

The implications of this perspective are particularly relevant for curriculum designers, language teachers, and teacher educators. Language programs informed by GELT can foster more psychologically safe classrooms, reduce affective barriers to communication, and better prepare learners for global, multilingual environments. Practitioners are encouraged to adopt inclusive assessments, diversify instructional models, and foreground learner agency as part of a holistic approach to language development.

Eventually, GELT offers a timely and necessary reimagining of English language education. As English continues to evolve beyond its traditional boundaries, language teaching must evolve with it—not only in what it teaches, but in how it supports the people learning it. Addressing learners’ psychological well-being is not ancillary to language acquisition; it is central. The GELT for psychological benefits framework provides a foundation for this shift, opening pathways for more resilient, creative, and empowered English users around the world.

## Data Availability

The original contributions presented in the study are included in the article/supplementary material, further inquiries can be directed to the corresponding author.
